# On the Evaluation of the Hyperthermic Efficiency of Magnetic Scaffolds

**DOI:** 10.1109/OJEMB.2023.3304812

**Published:** 2023-08-14

**Authors:** Matteo B. Lodi, Antonios Makridis, Konstantina Kazeli, Theodoros Samaras, Makis Angelakeris, Giuseppe Mazzarella, Alessandro Fanti

**Affiliations:** Department of Electrical and Electronic EngineeringUniversity of Cagliari 3111 09123 Cagliari Italy; Nanostructure Characterization: Technology and ApplicationsCIRI-AUTH 57001 Thessaloniki Greece

**Keywords:** Heating evaluation protocol, hyperthermia, magnetic biomaterials, specific absorption rate

## Abstract

*Goal:* Deep-seated tumors (DST) can be treated using thermoseeds exposed to a radiofrequency magnetic field for performing local interstitial hyperthermia treatment (HT). Several research efforts were oriented to the manufacturing of novel biocompatible magnetic nanostructured thermo-seeds, called magnetic scaffolds (MagS). Several iron-doped bioceramics or magnetic polymers in various formulations are available. However, the crucial evaluation of their heating potential has been carried out with significantly different, lab specific, variable experimental conditions and protocols often ignoring the several error sources and inaccuracies estimation. *Methods:* This work comments and provides a perspective analysis of an experimental protocol for the estimation methodology of the specific absorption rate (SAR) of MagS for DST HT. Numerical multiphysics simultions have been performed to outline the theoretical framework. After the in silico analysis, an experimental case is considered and tested. *Results:* From the simulations, we found that large overestimation in the SAR values can be found, due to the axial misplacement in the radiofrequency coil, while the radial misplacement has a lower impact on the estimated SAR value. *Conclusions:* The averaging of multiple temperature records is needed to reliably and effectively estimate the SAR of MagS for DST HT.

## I. Introduction

**Fig. 1.**
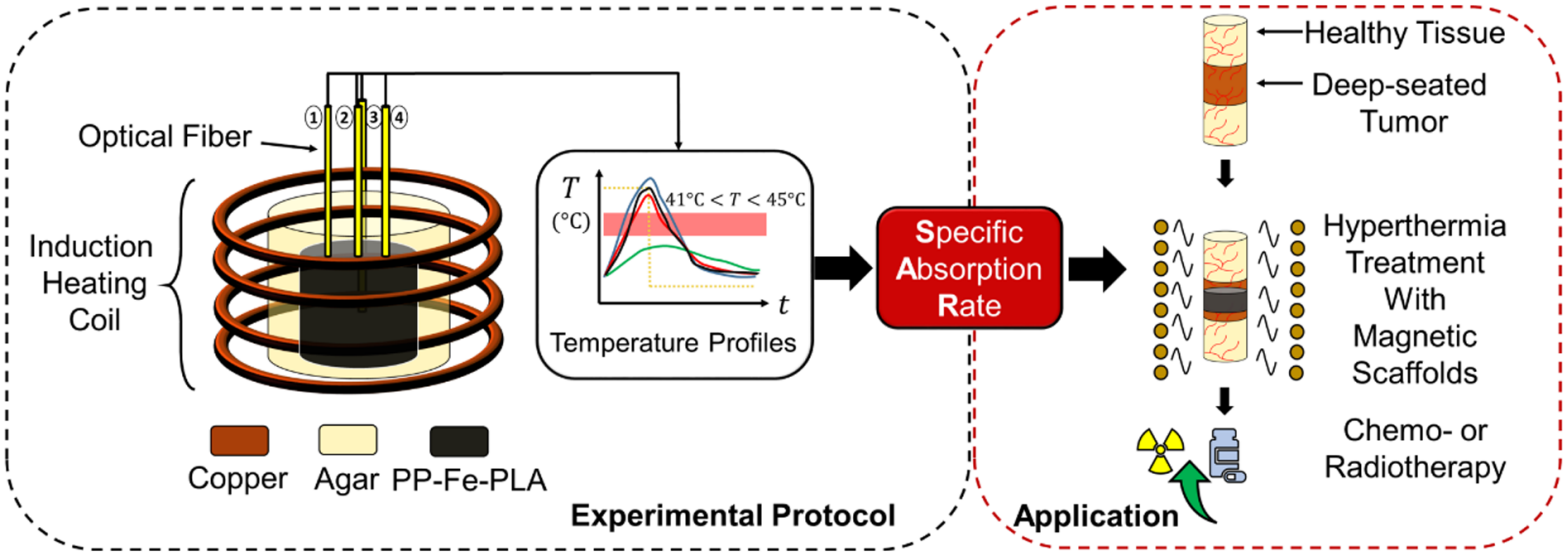
Graphical representation of the proposed experimental protocol for assessing the specific absorption rate (SAR) of biocompatible magnetic scaffolds (MagS) to be used as implants for local, interstitial hyperthermia treatment (HT) of deep-seated tumors (DST).

Cancer is a worldwide leading cause of death [1]. Malignant deep-seated tumors (DST - Fig. 1), such as cervix, colorectal, bladder carcinomas, brain malignancies or bone tumors, represent a class of neoplasms with peculiar clinical features [2], [3], [4]. The treatment of neoplasms grown in principal body cavities (e.g., abdomen, thorax, and skull) and deep sites is still challenging and demand for tumor- or patient-specific interventional strategies [4], [5]. DST gold standard treatments are surgery, radiotherapy and chemotherapy [5]. Despite the progresses, complementary techniques, such as immunotherapy [3], [5], have been investigated to control local recurrence rate, increasing the survival rate or enhance the quality of life. An emerging therapeutic strategy is the hyperthermia treatment (HT). HT proved to synergistically and significantly enhance the effectiveness of existing interventional strategies, such as chemio- and radiotherapy [2], [3], [4]. HT is a thermal therapy which aims to raise the temperature of a target tissue to 41–45 $^{\circ }$C for, at least, 30–60 min. This temperature range elicits the immune system response, increases the cytotoxicity and alters the tumor microenvironment. Furthermore, it increases the permeability to chemotherapeutics and enhance the sensitivity to radiation therapy [3], [4], [5], [6], [7], [8]. The therapeutic heat can be administered to the DST through ultrasounds (US) [9], photothermal (PT) devices [10] and electromagnetic (EM) energy. However, US and PT approaches present technological issues, e.g., limited penetration depth, and are ineffective in targeting DST. EM energy has the advantages of being noninvasive, contacless and highly controllable. Hence, EM energy has been adopted in clinical practice to deliver HT to DST through apacitive electrodes (8, 13.5 or 27.12 MHz) or by using arrays of antenna systems (433, 915 or 2450 MHz) [11]. Despite showing interesting clinical perspectives, EM-based HT strategies require focusing techniques, optimization approaches and complex instrumentation [3], [4], [7], [12].

Therefore, different heat administration modalities in HT DST have been investigated. The treatment of colo-rectal cancers with ferromagnetic implants has been previously studied [13], [14], [15]. Ferromagnetic implants made of NiCu and PdCo alloy with low Curie temperature have been proposed [16]. In [17], the size and recurrence of liver neoplasms was managed with stainless steel thermoseeds, whilst in [18] nickel-copper thermoseeds against melanoma have been investigated. Recently, a spherical ferromagnetic composite implant for tumor bed hyperthermia has been numerically and experimentally characterized [19]. However, these metallic thermoseeds present some major drawbacks, namely the safety for magnetic resonance imaging (MRI), the biocompatibility and long-term stability issues. Furthermore, the heat is administered through the eddy currents dissipation mechanism, thus requiring relatively high working frequencies $f\in [1-100]$ MHz [13], [14], [15].

Given the biocompatibility and post-operative, post-treatment aspects intrinsic of DST, ferromagnetic metal implant have been disregarded for the search of novel thermoseeds. To overcome such limitations, magnetic particle hyperthermia (MPH) for performing HT at these challenging tumor sites selectively has been proposed to treat regional and intra-tumoral regions with minimum intervention [20], [21], [22]. However, despite the expectations and potential of MPH, several aspects such as administration, treatable target volume, thermometry issues, potential toxicity and stability hinder its possible application against DST [23], [24], [25], [26].

The developement of new devices based on magnetic particles has been investigated. These solutions have been analyzed to maximize the heat delivered at the surgical bed site and control the local recurrence, while providing mechanical support to the injured tissue and provide a scaffold healthy tissue re-growth. So, the research focused on the development of multifunctional, theranostic platforms obtained by loading or doping an artificial tissue or a scaffold (bioceramic or polymeric) using magnetic particles (ferri-, ferro- or superparamagnetic) [27], [28], [29], [30], [31], [32], [33], [34], [35], [36], [37], [38]. Indeed, these magneto-responsive thermoseeds, called magnetic scaffolds (MagS), allow to perform the HT using a magnetic field in the kHz-range, after their implantation (Fig. 1). Then, following HT combined with chemio- or radiotherapy, MagS ensures a post-operative mechanical and biomimetic support for damaged tissue repair [27], [28], [29], [30], [31], [32], [33], [34], [35], [36], [37], [38]. The research is ongoing in this field. For instance, recently, a 3D printed superparamagnetic polymeric stent for treating tumors occurring in hollow organs was proposed and preliminary characterized [39]. In [40] poly-caprolactone (PCL) scaffolds have been drop-casted using iron oxide nanocrystals to obtain MagS for bone tumor treatment.

To push the forefront towards new clinical landscapes, a more definitive takeoff of using MagS for the DST HT is required. To this end, it is fundamental that MagS must satisfy the minimum quality assurance requirements of the interstitial hyperthermia treatment [49]. To treat DST, a given MagS should be capable of increasing the system temperature to the therapeutic level of 41–45 $^{\circ }$C and deposit a given amount of power per unit of mass (W/g) in the tumor volume in response to an alternating current (AC), radiofrequency (RF) magnetic field [11], [49]. The quantification of MagS hyperthermic potential is mandatory. When evaluating the hyperthermic efficiency we refer to the ability of a system to achieve hyperthermic conditions. So far, the heating efficiency of MagS has been reported in terms of the temperature increase ($\Delta T$) and time to reach it ($\Delta t$) [28], [33], [41]. However, in HT and, especially in MPH, the standardized and common metric of Specific Absorption Rate (SAR) has been adopted [49]. A discussion is in order. SAR is a measure of the energy, per unit time, absorbed by the target volume surrounding the thermoseed. Often, for MPH, the specific loss power (SLP) is considered. Even though these figure of merits have the same units (W/g), the SLP quantity describes the power achievable per gram of magnetic element in the material and refers to the energy dissipated by the MNPs to the system [50]. For MagS the adoption of SLP is not relevant, whilst the SAR as figure of merit is disregarded, or SAR has been evaluated with methodologically inappropriate means. As shown in Fig. 1, the way to quantify the heating response of a MagS is distinctly different from a typical MPH setup [50]. A different framework is needed. The proper setup to provide reliable heating evaluation will be rigorously explained and evaluated here. In the experimental estimation of the SAR several contributions must be considered. The factors affecting the SAR measurements are i) the AC magnetic field parameters (i.e. amplitude and frequency), ii) the exposure system features, iii) the thermometric aspects, but also iv) the MagS properties (e.g., the type of magnetic particle, their volume fraction, aggregation degree), as well as the biomaterial geometry and architecture. As a result, the comparison of different MagS is challenging. Recently, in [51], the influence of the geometry of a 3D-printed ferromagnetic MagS (i.e., pore size, distribution, porosity) on the HT performances have been investigated using three different experimental setups. While finding that architectural features of the MagS can impact on HT and heat diffusion, noticeable differences between calorimetric measurements carried out in air, deionized water and agar phantoms have been reported [50], [51]. Moving form these findings and considering the approach from [52], in [50] an accurate standardization protocol for determine the heating efficiency of 3D-printed MagS has been studied. In the current work, relying on an extensive analysis of the literature about the characterization of MagS for HT of DST, a thorough numerical study is carried out to identify the factors affecting the estimation of MagS SAR. Then, using ferromagnetic MagS samples [51], technical and methodological insights in the protocol proposed in [50] will be provided to drive an advanced characterization of the hyperthermic efficiency of MagS.

## II. Literature Review

Several MagS have been manufactured, such as the hadrystonite chemically doped with Fe ions [53], or the magnetic bioceramic for enhancing radiotherapy [54], and the Fe$_{3}$O$_{4}$-akermanite scaffold with self-catalytic activity [55]. In this framework, it is fundamental to provide a reliable way to compare these different biomaterials from the HT point of view. Despite the adoption of shared metrics, it is mandatory to manage and solve the measurement issues related to the heating efficiency. These tests are strongly dependent on the sample geometry and the measurement conditions (e.g., type of apparatus, field homogeneity, thermometry, etc.), but also on the estimation methodology. Indeed, the development, validity and effectiveness of procedure for evaluating the SAR of MagS is a highly underestimated aspect in the literature. Here, we will analyze the literature about MagS for the DST HT focusing on the experimental setups and methodologies used for evaluating their hyperthermic performances.

The calorimetric characterization of samples of poly-metilmetacrilate (PMMA) embedding Fe$_{3}$O$_{4}$ particles have been carried out by recording with an infrared camera (IR) the temperature of the saline-sample system exposed to an RF magnetic field [42]. In [42], Kexiao et al. considered different amount of saline volume (0.1 mL–0.2 mL), demonstrating a variability of $\pm 4.3^{\circ }$C from the maximum recorded temperature. Furthermore, issues in the repeatability due to the MagS sample placement inside the coil are evident (see Fig. 3, pg. 4198 from [42]). The misalignment of thermoseed implants to the external heating device is a relevant aspect for estimating the temperature and power losses [56]. For the P$_{2}$O$_{5}$-Fe$_{2}$O$_{3}$-CaO-SiO$_{2}$ ferromagnetic glass ceramic for bone tumor HT a 0.26 W/g SAR value has been estimated. The linear slope method has been used. The sample has been exposed to a 0.5 mT magnetic field, produced by a custom coil apparatus working at 100 kHz [43]. The HT potential characterization was carried out on a test tube, filled with deionized water. The temperature has been recorded every 60 s for 3 min with an optical fiber (OF) thermometer. The 2–3 mm in diameter and 0.9 g samples of apatite wollastonite, heat-treated glass ceramic (29% CaO, 31% SiO$_{2}$, 40% Fe$_{2}$O$_{3}$, 3% BzO$_{3}$, 3% P$_{2}$O$_{5}$ - wt%), synthesized by the group of Kokubo, have been implanted in rat tibial metaphysis and then characterized under the action of a 100 kHz, 30 mT external magnetic field produced by a C-type toroidal core of an induction generator [44]. The heating ability of the glass-ceramic implants has been assessed through fluoroptic thermometers (FT), recording a maximum temperature at the center of the thermoseed equal to 45 $^{\circ }$C after 50 min, resulting in an estimated SAR of 10 W/g. The temperature variation in the rat tibia was of about $\pm 5^{\circ }$C in the extra-cortical regions, indicating a large variability due to the temperature probe positioning [44]. Calorimetric measurements performed with a magnetic induction furnace on sol-gel calcium zinc iron silicon oxide samples have been performed in [45], [57]. The measurements have been carried out by placing 2 g of glass ceramic, with ferromagnetic ZnFe$_{2}$O$_{4}$ particles, in 20 ml deionized water in a quartz cuvette, positioned in the coil center. The temperature rise, recorded with a thermocouple (TC - 0.1 $^{\circ }$C resolution), starting from 24 $^{\circ }$C, ranged from a minimum 29 $^{\circ }$C to a maximum of 39 $^{\circ }$C in 3 min under the action of a 50 mT magnetic field working at 100 kHz. The estimated SAR varies in the range 5–9 W per gram of sample [45]. The 3D-printed, superparamagnetic PCL scaffolds embedding mesoporous bioactive glass (MBG) and magnetite nanoparticles (Fe$_{3}$O$_{4}$) have been manufactured and characterized as tissue substitutes, drug delivery systems and hyperthermia agents [46]. The magnetic MBG-PCL, when exposed to a 409 kHz and 18 mT magnetic field (DM100 System - nB nanoScale Biomagnetics, Spain) increased the temperature of 1 ml of water up to 60 $^{\circ }$C, starting from 20 $^{\circ }$C, for 8 min - 15 min of exposure [46]. The temperature has been recorded with an optical fiber, but the details about its placement and the effect on the measurement has not been evaluated. The intrinsic magnetic hydroxyapatite (HA) obtained by chemical doping could increase the temperature of distilled water of 40 $^{\circ }$C in 60 s, under the action of a 15 mT and 293 kHz magnetic field [41]. The magnetite loaded PCL scaffold from [41], under the same treatment parameter, reached only a 10 $^{\circ }$C increase, after 3 min. SAR values ranging from 5 to 30 W/g have been obtained for the weakly supeparamagnetic tri-calcium phosphate ($\beta$-Ca$_{3}$(PO$_{4})_{2}$) co-substituted with Fe$^{3+}$-Co$^{2+}$ ions thermo-seeds exposed to a 335 kHz and 13.5 mT magnetic field, in 1 mL of distilled water, for 40 min ($\Delta T$ of 12-22.5 $^{\circ }$C, starting from 26 $^{\circ }$C, measured with a copper TC) [47]. More recently, the calorimetric response of injectable graphite-modified Fe$_{3}$O$_{4}$-calcium phosphate bone cement scaffolds (9 mm x 4 mm blocks) from the work of Zhang et al. has been tested under the action of a 340 kHz, 10 mT magnetic field. SAR values of 18.7–30.2 W/g have been obtained from the temperature increase after 200 s, acquired by means of an infrared thermometer [48].

**Fig. 2.**
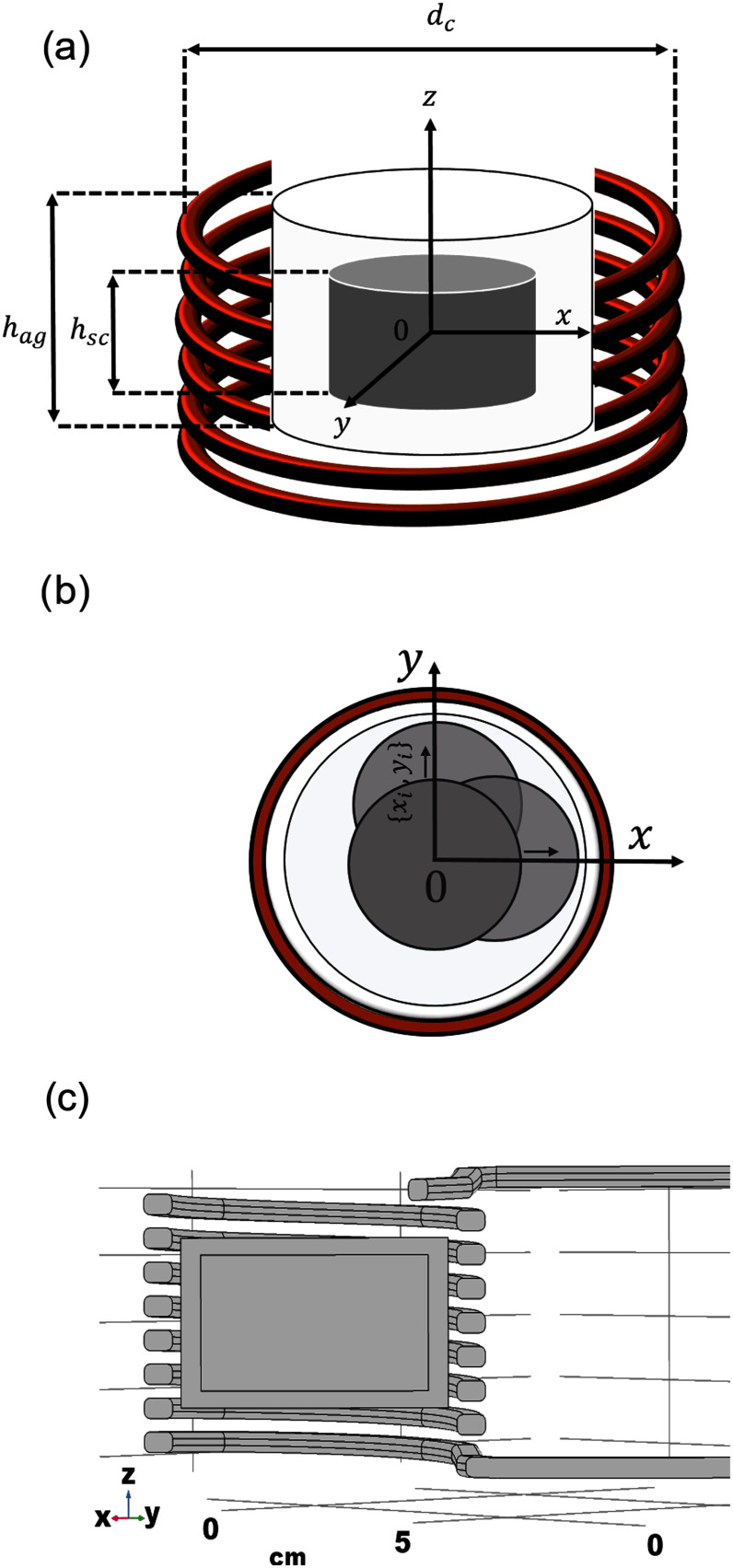
(a) System geometry for the numerical simulations of the different experimental setup for the calorimetric characterization of magnetic scaffolds (MagS). The heating system has conductors mad of copper, whilst the light grey cylinder represent the agarose matrix and the dark grey cylinder is the MagS. (b) Representation of the possible misplacement of the scaffold in the agar phantom. (c) Screenshot of the system geometry in Comsol Multiphysics v5.5 (Comsol Inc., Burlinghton, MA USA).

**Fig. 3.**
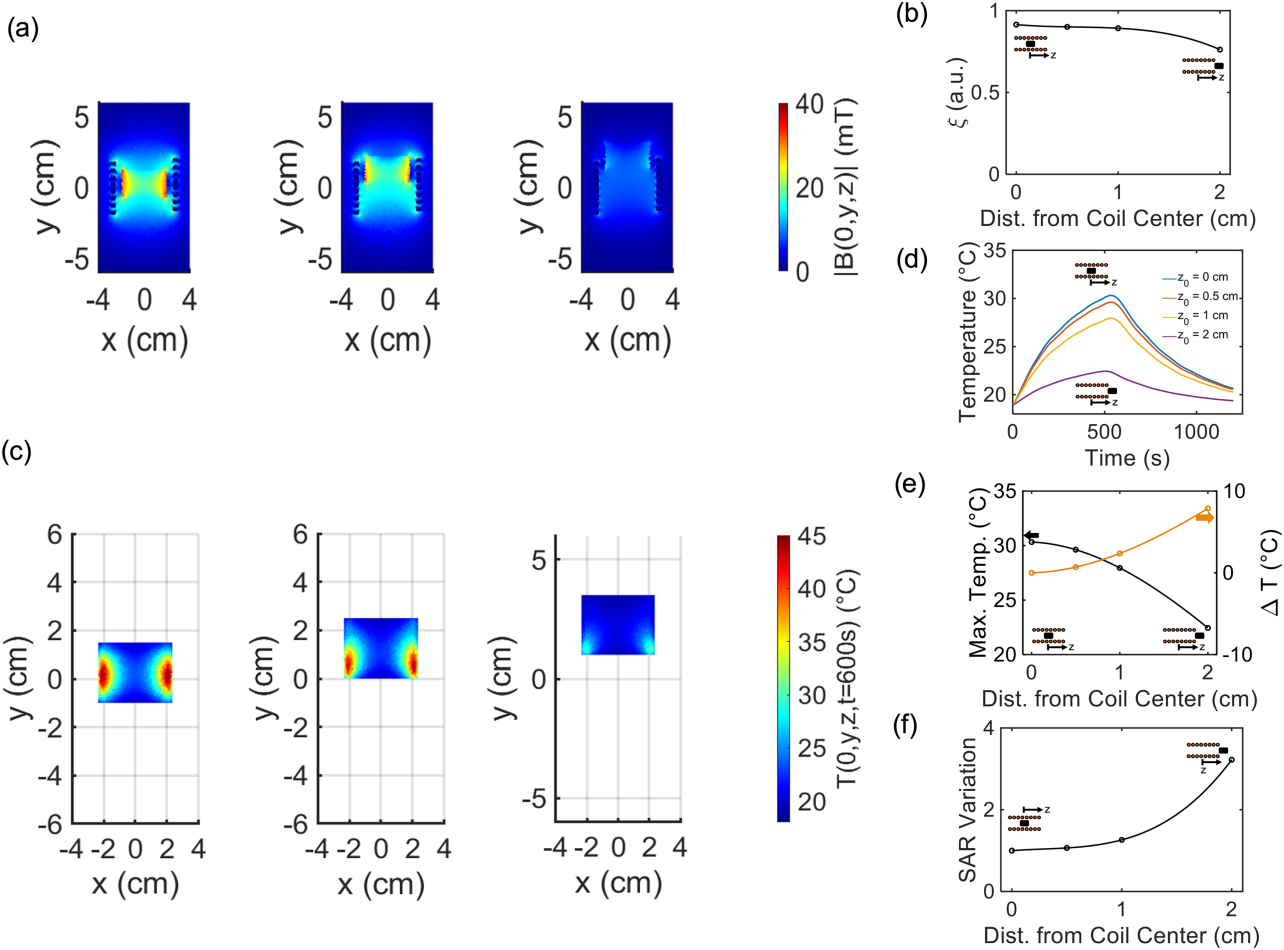
(a) Magnetic flux density distribution, in mT, for different position of the sample in the coil ($z_{0}=$0 cm, $z_{0}=$1 cm, $z_{0}=$2 cm). (b) Field homogeneity ($\xi$) for different sample placement. (c) Temperature distribution ($T(0,y,x$)), at $t=$600 s, for different position of the sample in the coil ($z_{0}=$0 cm, $z_{0}=$1 cm, $z_{0}=$2 cm). (d) Average magnetic scaffold (MagS) temperature versus time. (e) Peak temperatures, and relative differences between the initial condition for different coil axial positions. (f) Relative SAR variation, with respect to the initial condition, for different sample placement in the coil.

Nowadays, noticeable research efforts are spent for defining standardized protocols for characterizing the hyperthermic potential of magnetic ferrofluid for MPH [26], [52], [58], raising issues about inter-laboratory variations ($\sim$30% variation on the estimated SAR) and unveiling the various sources of uncertainties. This type of analysis and reasoning has not been carried out and applied to the experimental protocols for measuring the SAR of MagS. Indeed, by carefully looking at Table I, noticeable differences can be noticed in the characterization of MagS for HT. Very different working frequencies and field amplitudes have been used, as well as diverse thermometric approaches have been adopted. Also, the preferred media is deionized water, even though non-negligible convective contribution may influence the heat diffusion at the MagS interface [50], [51], [52]. Furthermore, it is worth stressing that, in the cited works, the external magnetic field is not turned off to investigate the effective heating and the thermal contribution of the background media (e.g., air, water, phantom or tissue) has not been taken into account properly [52]. Furthermore, SAR is not derived in most cases (Table I). Under these fuzzy experimental conditions, the effect of the variability of the experimental setups, combined with the neglection of uncertainty sources, is evident and results in SAR values in the range 0.2–30 W/g. This scenario is concerning and deserves a critical and engineering approach. With respect to the estimation of the SAR in the case of MPH, for MagS the situation is at an early stage and must be tackled immediately in order to set the limits, regulations and validity criteria for quantifying the hyperthermic potential of these innovative medicine tools for the HT of DTS.

**TABLE I:** State-of-The-Art Analysis of Calorimetric Characterization of Magnetic Scaffolds

**Biomaterial**	**Magnetic Phase**	$f$ (kHz)	$|B_{0}|$ (mT)	$\Delta T$ ($^{\circ }$C)	$T_{0}$ ($^{\circ }$C)	$\Delta t$ (min)	**Thermometry**	**Medium**	**SAR**	**Ref.**
PP	Fe $_{3}$ O$_{4}$	270	3.7	16	-	15	IR	Bile duct	-	[39]
HA	Fe $_{2}$ O$_{3}$, Fe $_{3}$ O$_{4}$	293	15	35	25	0.5	OF	Deionized water	-	[41]
Gelatin	Fe $_{3}$ O$_{4}$	293	15	17.5	-	1	OF	Deionized water	-	[41]
PCL	Fe $_{3}$ O$_{4}$	293	10	-	-	5	OF	Deionized water	-	[41]
PMMA	Fe $_{3}$ O$_{4}$	-	-	70	25	3	IR	Saline	-	[42]
P $_{2}$ O$_{5}$-CaO-SiO$_{2}$	Fe $_{2}$ O$_{3}$	100	0.5	16	15	16	OF	Deionized water	0.2	[43]
CaO-SiO $_{2}$-BzO$_{3}$-P$_{2}$ O$_{5}$	Fe $_{2}$ O$_{3}$	100	30	15	32	50	FT	Rat Tibia	10	[44]
Ca-SiO$_{2}$	ZnFe $_{2}$ O$_{4}$	100	50	27-39	20	3	TC	Deionized water	6.5-9	[45]
MBG-PCL	Fe $_{3}$ O$_{4}$	409	18	60-40	20	8-15	OF	Deionized water	1.4-4.7	[46]
Ca$_{3}$(PO$_{4})_{2}$	Fe $_{2}$ O$_{3}$, Fe $_{3}$ O$_{4}$	335	13.5	12-22.5	26	40	TC	Deionized water	-	[47]
Ca$_{3}$(PO$_{4})_{2}$	Fe $_{3}$ O$_{4}$	340	10	-	-	$\sim$3	IR	Air	18.7-30.2	[48]

(FT = Fluoroptic Thermometer, IR = IR Camera, of = Optical Fiber, TC = Thermocouple).

## III. Numerical Study

Given the lacks and flaws highlighted in reviewing the literature, in this work, we will perform numerical simulations and experiments to investigate the source of errors, the factors of influence and the most relevant aspects in the characterization of the hyperthermic potential of MagS for HT of DTS.

### A. Numerical Model

The finite element method (FEM) commercial software Comsol Multiphysics v5.5 (Comsol Inc., Burlinghton, MA USA) has been used to simulate the experimental setup proposed in [50], [51] for the SAR measurements and analyze it in detail (Fig. 1). The geometry for the simulation is shown in Fig. 2. A homogeneous cylindrical scaffold having magnetic permeability $\mu _{r}$, dielectric permittivity $\epsilon _{r}$ and elecrtical conductivity $\sigma$ has been considered. In this work we will consider a homogeneous geometry, even though MagS architectures are porous structures that can present complex geometry and variable tortuosity. The cylinder has diameter $d_{ag} = 2$ cm and height $h_{ag} = 2$ cm. The thermoseed is placed in an agar phantom ($h_{ag} = 4$ cm, $d_{ag} = 2.5$ cm), as shown in Fig. 2.

The induction heating coil is a single layer coil having $N=8$ turns. The coil is excited with a sinusoidal current ($I_{exc}$), working at $f = 400$ kHz, which is turn on at $t=0$, and turned-off at specific time $t_{off}$.

The calorimetric measurements is governed by the Ampere's law, in the time-harmonic fields formulation, so that

\begin{align*}
\nabla &\times \mathbf {H} = \mathbf {J} \tag{1}\\
\mathbf {E} &= -j \omega \mathbf {A} \tag{2}\\
\mathbf {J} &= \sigma \mathbf {E} + j \omega \mathbf {D} + \mathbf {J_{e}} \tag{3}\\
\nabla & \times \mathbf {A} = \mathbf {B} \tag{4}
\end{align*}

where $\mathbf {A}$ is the magnetic vector potential, $\mathbf {H}$ is the magnetic vector (Am $^{-1}$), $\mathbf {E}$ is the electric field vector (Vm$^{-1}$), whilst $\sigma$ is the electrical conductivity (in S$\cdot$ m$^{-1}$), $\mathbf {J}$ is the current density (A $\cdot$ m$^{-2}$). The term $\mathbf {D}=\epsilon _{0} \epsilon _{r} \mathbf {E}$ is the electric displacement field (C $\cdot$ m$^{-2}$), being $\epsilon _{0}$ the vacuum dielectric permittivity. In (1)–(4), $\mathbf {J_{e}}$ is the external current density (A $\cdot$ m$^{-2}$). The AC/DC module, in particular the Magnetic Field interface, has been used to solve (1)–(4). Once the magnetic field distribution has been computed, we evaluated the field homogeneity ($\xi$) in the MagS volume by calculating

\begin{equation*}
\xi = \frac{ m_{\mathbf {H}} }{ s_{\mathbf {H}} } \tag{5}
\end{equation*}

where $m_{\mathbf {H}}$ and $s_{\mathbf {H}}$ are the mean value and the standard deviation of the magnetic field in the MagS sample volume.

The key aspect during SAR mesurements is the heat dissipation of MagS. Depending on their magnetic features different mechanisms hold. A clarification is in order. In MagS, the embedded magnetic particles are the main elements responsible for the heat dissipation. Magnetic particles are known to dissipate heat via different independent mechanisms, i.e. eddy currents, hysteresis loss, Brownian relaxation and Neél relaxation. The eddy currents thermal energy conversion can be neglected [52]. On the other hand, hysteresis losses are due to the shift of magnetic domain walls in multi-domain materials and occurs in magnetic particles with a diameter higher than 200 nm. For smaller particles, if the magnetic anisotropy of the particle is higher than the viscous resistance of the surrounding medium, then the particle can rotate and generate heat due to the shear with the carrier fluid. However, for the case of MagS, the magnetic particles are embeded in a solid or very viscous matrix, and hence the Brownian relaxation process would be negligible or completely blocked, as already studied in [59]. Finally, for smaller magnetic particles ($\sim$15 nm), is the Neél mechanism that can be relevant. In this case, the heating is achieved by the dissipation due to the rearrangement during the rotation of the particle magnetic moment against its energy barrier [21]. From Table I, several MagS present ferromagnetic response. Therefore, in this work, we will focus on a ferromagnetic MagS. The exposure of a ferromagnetic scaffold to a time-varying magnetic field determines a dissipated power ($P_{m}$ in Wm$^{-3}$) to be computed as

\begin{equation*}
P_{m} = f A_{hyst} \tag{6}
\end{equation*}

where $A_{hyst}$ is the hysteresis loop area of the MagS, scaled by the specific density of the sample. Therefore, the $\mathbf {BH}$ curve, derived from static magnetic measurements, has been inserted in Comsol, interpolated linearly and used in the simulations [51]. Equation (6) is proportional to the mass of MagS sample. A remark is in order. For some of MagS reported in Table I, superparamagnetic nanoparticles have been used to functionalize the biomaterial and create a thermoseed. The proposed model can be applied to these systems by simply modifying (6) and substituing a suitable model for the power dissipation, as done in [60].

In this work, we will focus on a ferromagnetic polymeric scaffold, which will be considered as a case study. For this kind of MagS, the term $P_{m}$ is higher than the power dissipated due to the eddy currents in the ferromagnetic and in the agar material ($P_{e}=\frac{1}{2} \sigma |\mathbf {E}|^{2}$). However, the dielectric heating in the phantom cannot be neglected, being relevant to the measured temperature and the estimated SAR. Therefore, to investigate the heat transfer phenomena during the characterization of the heating efficiency of MagS, the following transient heat transfer equation has been solved

\begin{equation*}
\rho C_{p} \frac{\partial T}{\partial t} = \nabla \cdot (k\nabla T) - \mathbf {u} \cdot \nabla T + P_{e} + P_{m} \tag{7}
\end{equation*}

where $\rho$ is the material density (kg $\cdot$ m$^{-3}$), $C_{p}$ is the specific heat capacity (J $\cdot$ kg$^{-1}$K $^{-1}$), and $k$ is the thermal conductivity (W$\cdot$ m$^{-2}$K $^{-1}$). In (7), the term $\mathbf {u} \cdot \nabla T$ is the heat flux due to convection, for which $\mathbf {u}$ is a velocity (in m $\cdot$ s$^{-1}$). This term can be approximated through suitable boundary condition for air-solid interfaces, but cannot be neglected for heat transfer phenomena occuring in some fluids, such as water [50], [51], [52]. Therefore, in this work, to study also the different experimental setup and measurement environment found in the literature (Table I), we simulated the cases of MagS tested in air and in distilled water. More details about the simulations are given in the Supplementary Material (Supp. Mat.). The physical properties of the samples are reported in Table II. Equation (7) is solved assuming continuity of temperature and heat flux. At the agar-air interface the heat exchange is ruled by convection, so that

\begin{equation*}
-\mathbf {n}\cdot \nabla T = h_{a}(T-T_{a}) \tag{8}
\end{equation*}

where $\mathbf {n}$ is the normal vector and $T_{a} = 19\, ^{\circ }$C. The value of the heat transfer coefficient ($h_{a}$) reported in [51] has been used. The system was considered to be in thermal equilibrium at $t=0$ and a homogenous initial temperature distribution $T_{a} \, \forall \, x,y,z$ has been assumed. The Heat Transfer in Solid interface has been used.

**TABLE II:** Material Properties

**Material**	$\epsilon _{r}$	$\sigma$	$k$	$C_{p}$	$\rho$	**Ref.**
		(Sm$^{-1}$)	(W $\cdot$ m$^{-2}$K$^{-1}$)	(J $\cdot$ g$^{-1}$K$^{-1}$)	(g $\cdot$ cm$^{-3}$)	
**Air**	1	0	-	-	-	[60]
**Agar**	70	0.2	0.48	4.2	1	[62], [63]
**Copper**	-	5$\cdot 10^{7}$	-	-	-	[60]
**PLA**	2.5	10$^{-6}$	0.29	1.8	0.9	[64]
**PP**	3	10$^{-5}$	0.47	1.2	2.7	[65], [66], [67]

The simulated temperature field is used to compute the SAR as [50], [51], [52]

\begin{equation*}
\text{SAR} = C_{p} \frac{\Delta T}{\Delta t} \tag{9}
\end{equation*}

where $C_{p}$ is the specific heat is that of the agarose phantom, as taken from [61], [62], [63]. The term $\frac{\Delta T}{\Delta t}$ is the initial slope of the heating curve.

The MagS was assumed to be placed in the middle of the coil ($\mathbf {r_{0}}=\lbrace x=0,y=0,z=0\rbrace$, depoloyed at half of the coil height, i.e., $h_{C}/2$), initially. Then, we numerically investigated how the scaffold positioning in the induction heating system could affect the maximum temperature and the estimated SAR. In this framework, we considered two scenarios. We moved the MagS along the $z$-axis of the system up to 2 cm from its initial position, while leaving unchanged its radial position. Then, we simulated the possibility that the scaffold position varies in the $xy$-plane by varying the sample center coordinates ranging from $-3$ mm to 3 mm with a 1 mm step.

### B. In-Silico Findings

In this work we numerically investigated the experimental setups used for measuring the SAR of MagS. As reported in Table I the sample can be placed in different environments and media. Under these conditions, adequate thermometric methods should be implemented. However, some limitations, source of errors and potential shrotcomings exists and must be considered. We simulated the cases of MagS placed in-air and in-distilled water for measuring their SAR, as described in the Supp. Mat.

For the in-air case, which is the easier setup to implementi in a laboratory, the sample is placed in an induction coil and exposed to the magnetic field. The temperature can be recorded using an IR camera. As a matter of fact, only surface temperature ($T_{s}$) is acquired and can be post-processed to derive the SAR. By comparing the average surface temperature and the average volume temperature of a homogeneous cylindrical MagS exposed to a 30 mT and 400 kHz, a $\sim 10^{\circ }$C difference in the peak temperature exists (see Suppl. Mat.). Therefore, the SAR values which could be derived for the in-air experimental setup cannot be considered to be reliable. Hence, the results and the method reported in [48] cannot be used and applied. Furthermore, MagS are designed to present an architecture with a high interconncted porosity ($\sim \text{50--80}$%). The temperature patterns and the heat diffusion in the pores contributes to the heating in the surroundings [51]. Hence, for the in-air setup and IR thermometry, the role of the MagS porosity on the heat transfer could not be fully elucidated and properly accounted in the SAR estimation.

As regards the methodologies and the findings from [43], [45], [46], [47], in which the MagS are placed in saline or deionized water, a discussion is in order. During the RF heating of the MagS placed in water, the presence and action of convective motion due to the temperature gradients and local variation of the fluid physical properties must be considered, Indeed, neglecting the aspects related to sample deployement and the issues linked to the temperature probe placement, by numerically studying the average-temperature of the MagS in presece of natural convection lead to $\sim\! 4$ °C difference in the peak tempeature, while affecting the cooling rate (see Supp. Mat.). The non-linear effects of this natural convection on the heating and cooling dynamics cause this measurement setup to be difficult to implement, since the control of its conditions is not trivial. In this framework, the use of different temperature recording strategies (e.g., OF or TC- Table I) further stress the need of a standardized setup in the measurements MagS SAR.

Moving from these findings and considerations, in [50], [51] it has been proposed to place the MagS in an agarose phantom, expose the sample to the RF magnetic field for heating it and then derive the SAR. Indeed, an agarose matrix can mimic the cellular environment, while providing a flexible option for calorimetric measurements [50]. Despite solving some potential critical aspects and shortcomings of the in-air and in-water setups, the in-agar SAR tests have not been deeply investigated in [50], [51]. Indeed, methodological details such as sample placement and the homogeneity of the magnetic field have never been considered.

As regards the numerical study of the MagS sample misplacement, in this study we considered two situations. A potential source of error in the SAR values can be related to the placement of the sample along the coil axis. We simualted some RF heating experiments for which the MagS is placed is deployed in different $z$ positions. The findings are reported in Fig. 3. From Fig. 3(a), it can be noticed that as the sample is moved away from the initial position $z = 0$, the magnetic flux density values and the field homogeneity $\xi$ lower (Fig. 3(b)). Coherently, the strength and the uniformity of the magnetic field over the sample is lower, therefore the temperature distribution inside the agar phantom and the MagS lowers too (Fig. 3(c)), and asymmetries in the heating pattern arise. The average temperature in the MagS cylinder for the different cases of sample placement is shown in Fig. 3(d). The most remarkable features that depends on the axial distance from the coil center ($z$) is the peak temperature. In Fig. 3(e) it can be seen that, with respect to the sample placed in the center of the coil, more than 10 $^{\circ }$C of difference in the peak temperature can be recorded. As a matter of fact, considering (9), the different peak temperature would impact on the term $\Delta T$. Therefore, considering the ratio between the SAR at $z = 0$ and the other cases, since the $\Delta T$ decreases, the estimated SAR would be higher. Even though these findings may appear trivial, considering the methodology and results reported in [42], these results demonstrate that the sample placement is a key factor for an accurate estimation of the SAR. The best condition for evaluating the heating efficiency of the MagS is to expose them to a magnetic field as homogeneous as possible.

**Fig. 4.**
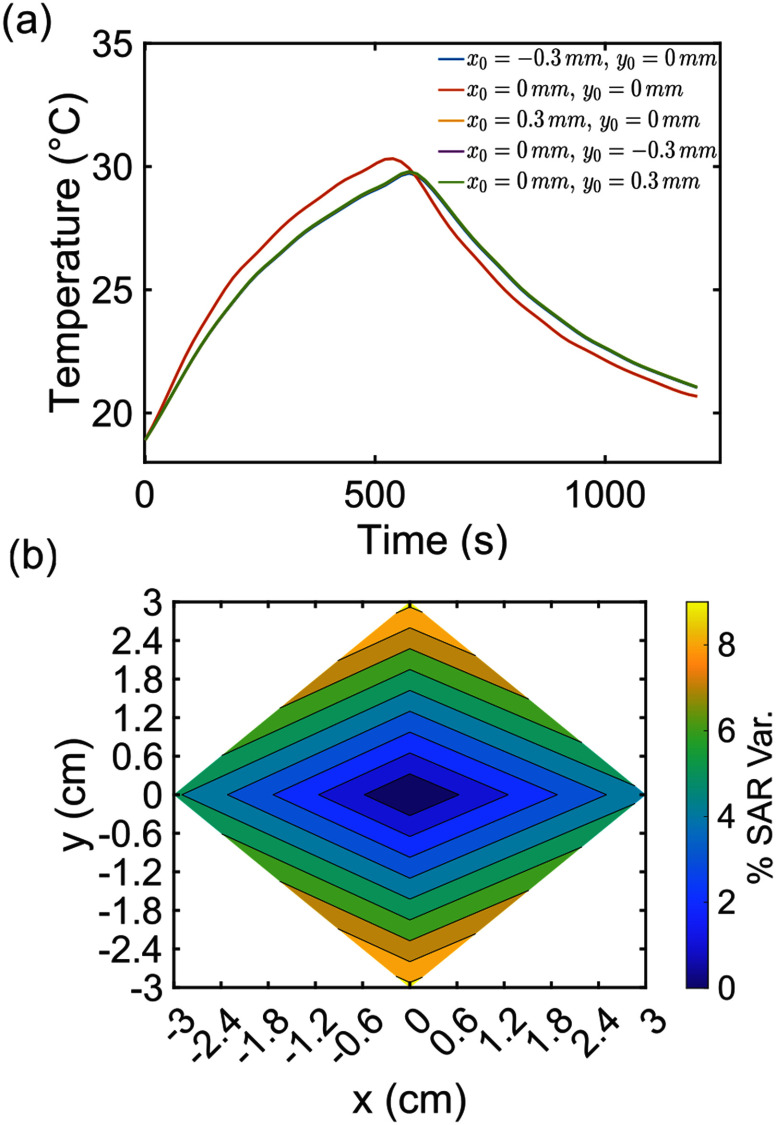
(a) Simulated average temperature of the magnetic thermossed as a function of time. There is a partial overlap of the curves. (b) Percentual variation of the SAR with respect to the values estimated for the scaffolds placed in $\lbrace x=0,y=0\rbrace$.

The previous simulations and in silico findings were derived considering a MagS placed perfectly centered in the coil ($x_{0}=0,\,,y_{0}=0$). However, it is likely that during the phantom preparation and the MagS incorporation, the sample can be placed incorrectly and could shift from the center. The homogeneity of the field would be slightly affected (in the measure of few % - see Supp. Mat.), but relevant effects on the estimated SAR could arise. Therefore, we simualated the SAR test for a MagS with initial position is moved away from the center in the range $x \in [-3,3]$ mm and $y \in [-3,3]$ mm. The findings are given in Fig. 4. Considering the simulated volume-averaged temperature curves, slight differences in the peak temperature can be noticed, as shown in Fig. 4(a). In particular, a maximum difference of 1 $^{\circ }$C has been found (see Supp. Mat.). However, less than 1 $^{\circ }$C of difference in the $\Delta T$ results in a large SAR variation (Fig. 4(b)). In terms of percentage variation, considering the initial position $x,y,z = 0$ as reference (see Fig. 2), the SAR can be overestimate up to the 8%. Of course, given these findings, a combination of axial and radidal displacemtne can be key factors in the drastic misestimation of the SAR.

The findings of our numerical simulations highlight that the sample placement is crucial and must be carefully considered before applying the proposed methodology to estimate the SAR of MagS. The numerical study is preliminary and the experimental part cannot be disregarded.

## IV. Experimental Setup

### A. Magnetic Scaffold: Architecture and 3D-Printing

The scaffold samples considered in this study are made of PolyLactic Acid (PLA) filament loaded with iron particles (Proto-Pasta, USA - PP herein), with a 40 wt.% content of microparticles having an average size of $\sim$40 $\mu$m. The scaffold architecture was created using Rhinoceros 7 (Mc Neel, Canada) modeling software, then the ideaMaker (Raise 3D Technologies, Inc., USA) was used to derive the stereolithography (STL) files and the g-code for printing them by using the Raise3D Pro2 Plus 3-D FDM (Fused Deposition Modeling) printer. The extrusion temperature was set to 210 $^{\circ }$C for the 0.45 mm nozzle, with a 100% infill density and a 50 mm $\cdot$ s$^{-1}$ printing speed. The bed temperature was kept at 45 $^{\circ }$C.

### B. Static Magnetic Measurements

The static magnetic properties of the ferromagnetic MagS have been characterized toto. In particular, the magnetic hysteresis loops of the iron-PLA have been acquired using an Oxford Instruments 1.2H/CF/HT Vibrating Sample Magnetometer (VSM), at the system temperature of 300 K and by varying the magnetic field strengths in the range $[-1,1]$ T.

The findings are reported in Fig. 6 and have been used to fed the numerical simulations.

**Fig. 5.**
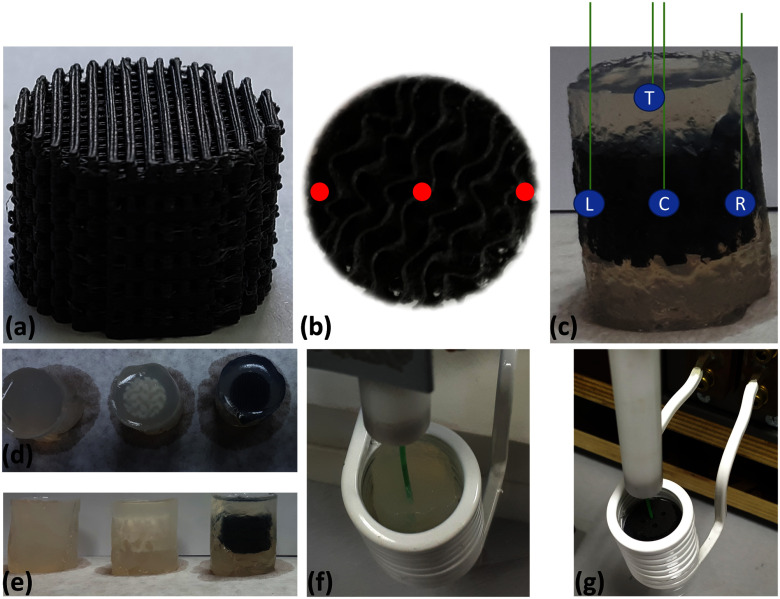
Preparation of (a) 3D printed magnetic scaffold (MagS) sample for magnetic hyperthermia experiment. (b) 3 symmetrical holes are opened to place the temperature probe-optical fiber in (c) three positions within the MagS and one background measurement. Control samples in agarose gel from (d) top and (e) side view. Heating evaluation experimental setup with (f) control and (g) MagS samples placed at the center of the induction heating coil with optical fiber (green).

**Fig. 6.**
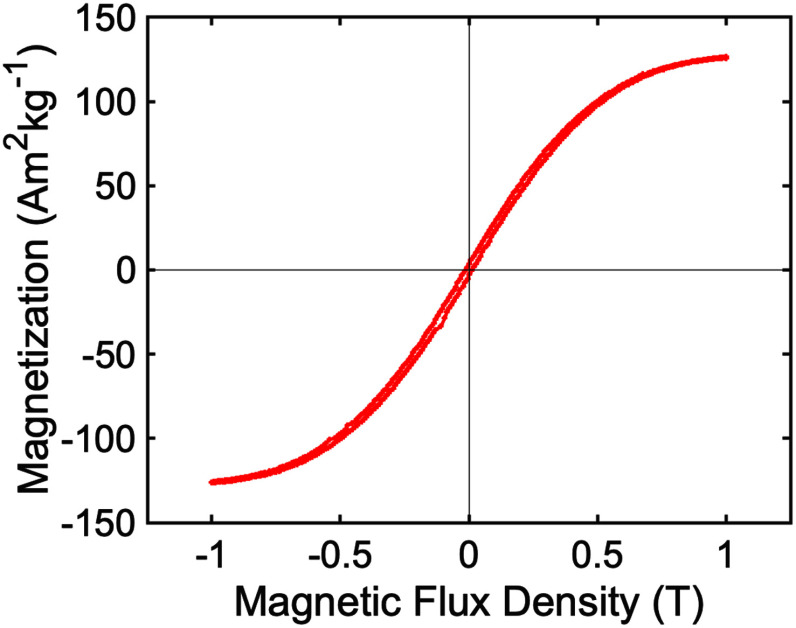
Measured magnetization (Am $^{2}$ kg$^{-1}$) of the ferromagentic poly-lactic acid (PLA) as a function of the applied magnetic flux density (T).

### C. Magnetic Hyperthermia Characterization

This work aims to specify the experimental steps on accurate measurement of heating efficiency of 3D printed MagS. To unravel this unexplored research field, an experimental protocol is depicted in Fig. 2. For 3D-printed MagS (Fig. 2(a)), conventional heating evaluation experimental approaches used on MNPs for MPH are not applicable. For this reason, a different methodological approach must be pursued. For MagS, three holes are opened from the top to the scaffold center, as shown with the red dots in Fig. 2(b). MagS are then placed in an agarose matrix to form a fluid surrounding mimicking the tissue environment and allowing the temperature probe (i.e., an optical fiber colored as green in Fig. 2(c), (f), (g)) to record the heat diffusion in four different positions (T - Top, L - Left, C - Center, and R - Right). The test gels have been prepared by mixing homogeneously, under magnetic stirring, the scaffolds with distilled water (50 ml in agarose concentration of 4 g $\cdot$ ml$^{-1}$), at room temperature. The concentrations of gelling agents was 5.0 wt%. Similar samples of 3D printed PLA scaffolds and only agarose matrix (Fig. 2(e)) are the non-magnetic reference samples in this experimental procedure (Fig. 2(g)).

Heating efficiency under magnetic hyperthermia conditions is evaluated through calorimetric measurements using the Easyheat AC field induction heating system, provided by Ambrell, operating at the power of 2.4 kW and at a frequency of 400 kHz. The $|\mathbf {H}|\times f$ product is 9.5$\cdot 10^{9}$ Am $^{-1}$ s$^{-1}$ [68]. The applied magnetic flux density field intensity is 30 mT. The sample is deployed centered in the 8-turn induction heating coil with optical fibre placed in the corresponding positions (Fig. 5(f) and (g)). The magnetic field strength inside the coil has been measured by using a small coil probe having diameter of 7.3 mm, 2.5 turns, 3 cm of height. The coil has been placed inside the induction heating coil and connected with an oscilloscope to measure the peak-to-peak value of the electromotive force (in V) and convert it to H(kA/m) through the use of Faraday's law. The SAR is then estimated using (9). All samples' initial temperature (before turning the magnetic field on) was set at 19 $^{\circ }$C, while coil cooling water temperature was kept stable during the procedure. Meanwhile, infrared (IR) camera (FLIR i3, FLIR Systems, USA) was used to have a global temperature recording of the experimental setup (i.e., not only sample's temperature), in order to control better possible non-magnetic heating losses and monitor the self-heating of the coil.

## V. Results

**Fig. 7.**
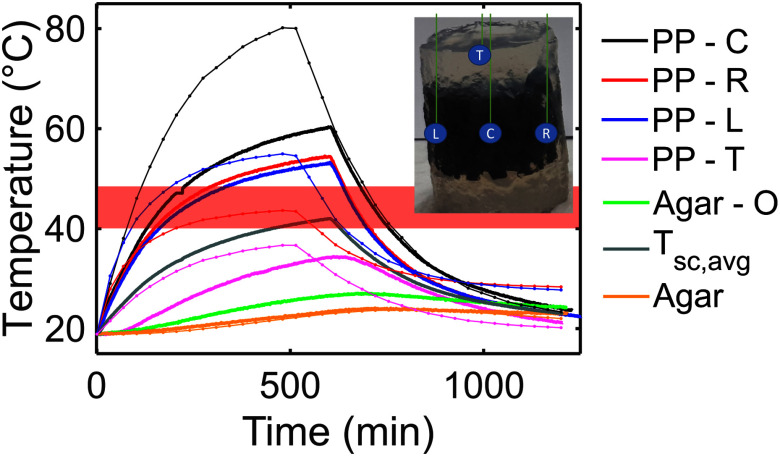
Expeirmental temperature curves acquired in four different position (T = Top, C = Center, R = Right, L = Left) in the magnetic scaffold (MagS) and in the agarose phantom (O = Out). The simulated counterparts are reported in lighter colors. The shaded red area represents the typical temperature range for hyperthremia treatment.

To evaluate the SAR of MagS the experimental setup shown in Fig. 5 and the methodology described in Section II.B have been used. The different temperature curves recorded during the magnetic hyperthermia test are shown in Fig. 7. From Fig. 7 it can be noticed that the highest temperatures are reached in the MagS center (C - black curve), as confirmed by the simulations. The difference between the simulated and measured values is due to the fact that, in silico, a homogeneous cylinder has been considered, whilst, in practice, MagS are heterogeneous and porous materials. The temperature dynamics in the measurement points R and L are quite similar between them, depsite the peak temperature is ∼2.5 °C lower than that measuered in the MagS center. The difference is due to the MagS architecture and its porosity, but also to the fact the probe is placed where the magnetic field is less homogeneous and the temperature is lower due to diminished deposited power (see Figs. 3 and 4). By relying on the findings form our numerical study, using only the temperature curves acquired in points L and R in (9) would result in a misleading estimation of the SAR value. Similarly, the temperature records at the MagS top (T - fuchsia curve in Fig. 7) and $\sim$1 cm outside the sample volume in the agar (O - green curve in Fig. 7) have a lower peak temperature value. This is due to the fact that lower values of magnetic field strenght and homogeneity are found at this location (see Fig. 3). Furthremore, for these probe locations, by observing the initial slope of the temperature vs. time curve, a clear decrease in the $\Delta T/\Delta t$ must be noticed (Fig. 7). The different heat diffusion regime strongly affects the sape of the curve and can strongly impact on the final estimated SAR value. These findings are of relevance for cases in which the MagS is made of fragile or very hard material and the probe cannot be inserted in the sample volume to properly investigate the heating dynamics in the biomaterial volume (Table I). Furthermore, the numerical and experimental findings shine a light on the variability of the results reported in Table I.

The considerations done for the temperature curves are fundamental for the SAR estimation. Indeed, if the temperature curve in the agar phantom is considered, an SAR of $\sim$1 W/g is found for the MagS considered in this study. On the other hand, if the temperature curves acquired inside the MagS (i.e., C, R, L, T) are used to compute an average SAR, so that (9) is modified in

\begin{equation*}
\text{SAR} = C_{p} \frac{1}{N_{T}} \sum ^{N_{T}}_{i=1}{ \frac{\Delta T_{i}}{\Delta t_{i}} } \tag{10}
\end{equation*}

a value of 1.2 $\pm$ 0.2 W/g is found. In (10), $N_{T}$ is the number of temperature curves, while the other symbols retain their usual meaning. This value is in agreement to the SAR value reported in [51] and [50] for MagS with similar architecture, weight and properties. Here, it is necessary to report a noticeable 20% SAR variation. As a matter of fact, from our analysis and from our findings, the SAR values reported in Table I could not be directly compared to the SAR values derived for our ferromagnetic PP scaffolds. Indeed, since the methodological details are missing for the MagS in Table I a fair comparison cannot be performed. Therefore, careful guidelines must be followed to obtain reliable and comparable results.

## VI. Conclusion and Discussion

This work deals with the investigation of the experimental parameters and estimation methodology on the SAR of MagS for DST HT. In particular, this work thoroughly investigates through extensive numerical simulations the factors affecting the reliability of the measurements of MagS SAR. The SAR measurements of MagS are affected by i) the sample placement in the coil, ii) the magnetic field homogeneity, the temperature probe placement, and iv) the biomaterial architecture. Furthermore, we specify an experimental procedure to perform accurate measurement of heating efficiency of 3D printed MagS to reduce the influence of probe placement and the biomaterial archietecture. To unravel this unexplored research field and foster the design, characterization and evaluation of MagS as hyperthremia agents, the proposed experimental protocol could be adopted, as shared methodology, for the assessment of the heating potential of any innovative thermoseeds for DST treatment. In particular, how framework could be employed to evaluate the heating performances of ferromagnetic or superparamagnetic MagS. Future works will deal with the numerical and experimental investigation of how the geometric and architecture parameters of the MagS can affect the quality of the heating.

## References

[1] K. D. Miller , “Cancer treatment and survivorship statistics 2016,” CA: A Cancer J. Clinicians, vol. 66, no. 4, pp. 271–289, 2016. 10.3322/caac.21349 27253694

[2] G. P. Skandalakis , “Hyperthermia treatment advances for brain tumors,” Int. J. Hyperthermia, vol. 37, no. 2, pp. 3–19, 2020. 32672123 10.1080/02656736.2020.1772512 PMC7756245

[3] I. Kuo , “Potential enhancement of host immunity and anti-tumor efficacy of nanoscale curcumin and resveratrol in colorectal cancers by modulated electro-hyperthermia,” BMC Cancer, vol. 20, no. 1, pp. 1–13, 2020. 10.1186/s12885-020-07072-0 PMC7324975 32600429

[4] N. R. Datta, H. P. Kok, H. Crezee, U. S. Gaipl, and S. Bodis, “Integrating loco-regional hyperthermia into the current oncology practice: SWOT and TOWS analyses,” Front. Oncol., vol. 10, 2020, Art. no. 819. 10.3389/fonc.2020.00819 PMC7303270 32596144

[5] M. Hurwitz and P. Stauffer, “Hyperthermia, radiation and chemotherapy: The role of heat in multidisciplinary cancer care,” Seminars Oncol., vol. 41, no. 6, pp. 714–729, 2014. 10.1053/j.seminoncol.2014.09.014 25499632

[6] A. Oei , “Molecular and biological rationale of hyperthermia as radio-and chemosensitizer,” Adv. Drug Del. Rev., vol. 163, pp. 84–97, 2020. 10.1016/j.addr.2020.01.003 31982475

[7] H. P. Kok , “Locoregional peritoneal hyperthermia to enhance the effectiveness of chemotherapy in patients with peritoneal carcinomatosis: A simulation study comparing different locoregional heating systems,” Int. J. Hyperthermia, vol. 37, no. 1, pp. 76–88, 2020. 10.1080/02656736.2019.1710270 31969039

[8] S.-Y. Lee, G. Fiorentini, A. M. Szasz, G. Szigeti, A. Szasz, and C. A. Minnaar, “Quo vadis oncological hyperthermia (2020)?,” Front. Oncol., vol. 10, 2020, Art. no. 1690. 10.3389/fonc.2020.01690 PMC7499808 33014841

[9] Z. Izadifar, Z. Izadifar, D. Chapman, and P. Babyn, “An introduction to high intensity focused ultrasound: Systematic review on principles, devices, and clinical applications,” J. Clin. Med., vol. 9, no. 2, 2020, Art. no. 460. 10.3390/jcm9020460 PMC7073974 32046072

[10] J. Wang , “Gold nanobipyramid-loaded black phosphorus nanosheets for plasmon-enhanced photodynamic and photothermal therapy of deep-seated orthotopic lung tumors,” Acta Biomaterialia, vol. 107, pp. 260–271, 2020. 32147471 10.1016/j.actbio.2020.03.001

[11] H. P. Kok , “Heating technology for malignant tumors: A review,” Int. J. Hyperthermia, vol. 37, no. 1, pp. 711–741, 2020. 32579419 10.1080/02656736.2020.1779357 PMC7781160

[12] D. Baskaran and K. Arunachalam, “Design and experimental verification of 434 MHz phased array applicator for hyperthermia treatment of locally advanced breast cancer,” IEEE Trans. Antennas Propag., vol. 69, no. 3, pp. 1706–1715, Mar. 2021.

[13] P. R. Stauffer , “Observations on the use of ferromagnetic implants for inducing hyperthermia,” IEEE Trans. Biomed. Eng., vol. BME-31, no. 1, pp. 76–90, Jan. 1984. 10.1109/TBME.1984.325373 6724613

[14] P. R. Stauffer, T. C. Cetas, and R. C. Jones, “Magnetic induction heating of ferromagnetic implants for inducing localized hyperthermia in deep-seated tumors,” IEEE Trans. Biomed. Eng., vol. BME-31, no. 2, pp. 235–251, Feb. 1984. 10.1109/TBME.1984.325334 6706353

[15] T. C. Cetas, E. J. Gross, and Y. Contractor, “A ferrite core/metallic sheath thermoseed for interstitial thermal therapies,” IEEE Trans. Biomed. Eng., vol. 45, no. 1, pp. 68–77, Jan. 1998. 9444841 10.1109/10.650354

[16] J. A. Paulus, J. S. Richardson, R. D. Tucker, and J. B. Park, “Evaluation of inductively heated ferromagnetic alloy implants for therapeutic interstitial hyperthermia,” IEEE Trans. Biomed. Eng., vol. 43, no. 4, pp. 406–413, Apr. 1996. 8626189 10.1109/10.486260

[17] B.-H. Park, B. S. Koo, Y. K. Kim, and M. K. Kim, “The induction of hyperthermia in rabbit liver by means of duplex stainless steel thermoseeds,” Korean J. Radiol., vol. 3, no. 2, pp. 98–104, 2002. 12087199 10.3348/kjr.2002.3.2.98 PMC2713842

[18] Q.-S. Xia , “Feasibility study of high-temperature thermoseed inductive hyperthermia in melanoma treatment,” Oncol. Rep., vol. 25, no. 4, pp. 953–962, 2011. 21234522 10.3892/or.2011.1143

[19] A. M. Osintsev , “Characterization of ferromagnetic composite implants for tumor bed hyperthermia,” IEEE Trans. Magn., vol. 57, no. 9, Sep. 2021, Art. no. 5400108. 10.1109/tmag.2021.3097915 PMC8443243 34538882

[20] Q. Pankhurst, N. Thanh, S. Jones, and J. Dobson, “Progress in applications of magnetic nanoparticles in biomedicine,” J. Phys. D: Appl. Phys., vol. 42, no. 22, 2009, Art. no. 224001.

[21] S. Dutz and R. Hergt, “Magnetic particle hyperthermia-a promising tumour therapy?,” Nanotechnology, vol. 25, no. 45, 2014, Art. no. 452001. 10.1088/0957-4484/25/45/452001 25337919

[22] A.-R. Tsiapla , “Mitigation of magnetic particle hyperthermia side effects by magnetic field controls,” Int. J. Hyperthermia, vol. 38, no. 1, pp. 511–522, 2021. 33784924 10.1080/02656736.2021.1899310

[23] W. J. Atkinson, I. A. Brezovich, and D. P. Chakraborty, “Usable frequencies in hyperthermia with thermal seeds,” IEEE Trans. Biomed. Eng., vol. BME-31, no. 1, pp. 70–75, Jan. 1984. 10.1109/TBME.1984.325372 6724612

[24] M. D. Nieskoski and B. S. Trembly, “Comparison of a single optimized coil and a Helmholtz pair for magnetic nanoparticle hyperthermia,” IEEE Trans. Biomed. Eng., vol. 61, no. 6, pp. 1642–1650, Jun. 2014. 24691525 10.1109/TBME.2013.2296231

[25] H. F. Rodrigues, G. Capistrano, and A. F. Bakuzis, “In vivo magnetic nanoparticle hyperthermia: A review on preclinical studies, low-field nano-heaters, noninvasive thermometry and computer simulations for treatment planning,” Int. J. Hyperthermia, vol. 37, no. 3, pp. 76–99, 2020. 10.1080/02656736.2020.1800831 33426989

[26] I. Rubia-Rodríguez , “Whither magnetic hyperthermia? A tentative roadmap,” Materials, vol. 14, no. 4, 2021, Art. no. 706. 10.3390/ma14040706 PMC7913249 33546176

[27] D. Bahadur and J. Giri, “Biomaterials and magnetism,” Sadhana, vol. 28, no. 3, pp. 639–656, 2003.

[28] A. Baeza, D. Arcos, and M. Vallet-Regí, “Thermoseeds for interstitial magnetic hyperthermia: From bioceramics to nanoparticles,” J. Phys.: Condens. Matter, vol. 25, no. 48, Nov. 2013, Art. no. 484003. [Online]. Available: https://doi.org/10.1088/0953-8984/25/48/484003 10.1088/0953-8984/25/48/484003 24200980

[29] Y. Li , “Magnetic hydrogels and their potential biomedical applications,” Adv. Funct. Mater., vol. 23, no. 6, pp. 660–672, 2013.

[30] H.-Y. Xu and N. Gu, “Magnetic responsive scaffolds and magnetic fields in bone repair and regeneration,” Front. Mater. Sci., vol. 8, no. 1, pp. 20–31, 2014.

[31] A. A. Adedoyin and A. K. Ekenseair, “Biomedical applications of magneto-responsive scaffolds,” Nano Res., vol. 11, no. 10, pp. 5049–5064, 2018.

[32] B. Smolková, M. Uzhytchak, A. Lynnyk, Š. Kubinová, A. Dejneka, and O. Lunov, “A critical review on selected external physical cues and modulation of cell behavior: Magnetic nanoparticles, non-thermal plasma and lasers,” J. Funct. Biomaterials, vol. 10, no. 1, 2019, Art. no. 2. 10.3390/jfb10010002 PMC6463085 30586923

[33] M. Miola , “Glass-ceramics for cancer treatment: So close, or yet so far?,” Acta Biomaterialia, vol. 83, pp. 55–70, 2019. 30415065 10.1016/j.actbio.2018.11.013

[34] J. Peng, J. Zhao, Y. Long, Y. Xie, J. Nie, and L. Chen, “Magnetic materials in promoting bone regeneration,” Front. Mater., vol. 6, 2019, Art. no. 268.

[35] S. S. Danewalia and K. Singh, “Bioactive glasses and glass–ceramics for hyperthermia treatment of cancer: State-of-art, challenges and future perspectives,” Mater. Today Bio, vol. 10, 2021, Art. no. 100100. 10.1016/j.mtbio.2021.100100 PMC7985406 33778466

[36] O. Sedighi, A. Alaghmandfard, M. Montazerian, and F. Baino, “A critical review of bioceramics for magnetic hyperthermia,” J. Amer. Ceram. Soc., vol. 105, no. 3, pp. 1723–1747, Mar. 2022.

[37] J. Liao, R. Han, Y. Wu, and Z. Qian, “Review of a new bone tumor therapy strategy based on bifunctional biomaterials,” Bone Res., vol. 9, no. 1, pp. 1–13, 2021. 33727543 10.1038/s41413-021-00139-z PMC7966774

[38] P. I. Soares, J. Romao, R. Matos, J. C. Silva, and J. P. Borges, “Design and engineering of magneto-responsive devices for cancer theranostics: Nano to macro perspective,” Prog. Mater. Sci., vol. 116, 2021, Art. no. 100742.

[39] B. Mues , “Nanomagnetic actuation of hybrid stents for hyperthermia treatment of hollow organ tumors,” Nanomaterials, vol. 11, no. 3, 2021, Art. no. 618. 10.3390/nano11030618 PMC7999083 33801426

[40] M. B. Lodi , “Influence of magnetic scaffold loading patterns on their hyperthermic potential against bone tumors,” IEEE Trans. Biomed. Eng., vol. 69, no. 6, pp. 2029–2040, Jun. 2022. 34882544 10.1109/TBME.2021.3134208

[41] M. Bañobre-López , “Hyperthermia induced in magnetic scaffolds for bone tissue engineering,” IEEE Trans. Magn., vol. 50, no. 11, Nov. 2014, Art. no. 5400507.

[42] K. Yu , “PMMA-fe3o4 for internal mechanical support and magnetic thermal ablation of bone tumors,” Theranostics, vol. 9, no. 14, 2019, Art. no. 4192. 10.7150/thno.34157 PMC6592182 31281541

[43] P. Ji, Y. Wang, M. Zhang, B. Li, and G. Zhang, “P$_{2}$O$_{5}$-Fe$_{2}$O$_{3}$-CaO-SiO$_{2}$ ferromagnetic glass-ceramics for hyperthermia,” Int. J. Appl. Ceram. Technol., vol. 15, no. 5, pp. 1261–1267, 2018.

[44] K. Ohura , “A heat-generating bioactive glass–ceramic for hyperthermia,” J. Appl. Biomaterials, vol. 2, no. 3, pp. 153–159, 1991. 10.1002/jab.770020303 10149080

[45] Y. Jiang, J. Ou, Z. Zhang, and Q.-H. Qin, “Preparation of magnetic and bioactive calcium zinc iron silicon oxide composite for hyperthermia treatment of bone cancer and repair of bone defects,” J. Mater. Sci.: Mater. Med., vol. 22, no. 3, pp. 721–729, 2011. 21221727 10.1007/s10856-010-4225-z

[46] J. Zhang , “3D-printed magnetic Fe_3_O_4_/MBG/PCL composite scaffolds with multifunctionality of bone regeneration, local anticancer drug delivery and hyperthermia,” J. Mater. Chem. B, vol. 2, no. 43, pp. 7583–7595, 2014. 32261896 10.1039/c4tb01063a

[47] R. K. Singh, M. Srivastava, N. Prasad, P. Shetty, and S. Kannan, “Hyperthermia effect and antibacterial efficacy of Fe_3_/Co_2_ co-substitutions in $\beta$-ca3 (po4) 2 for bone cancer and defect therapy,” J. Biomed. Mater. Res. Part B: Appl. Biomaterials, vol. 106, no. 3, pp. 1317–1328, 2018. 10.1002/jbm.b.33921 28661574

[48] K. Zhang, G. Li, Z. Pei, S. Zhao, A. Jing, and G. Liang, “Injectable graphite-modified Fe_3_O_4_/calcium phosphate bone cement with enhanced heating ability for hyperthermia,” Mater. Technol., vol. 35, no. 13/14, pp. 863–871, 2020.

[49] H. Dobšíček Trefná , “Quality assurance guidelines for interstitial hyperthermia,” Int. J. Hyperthermia, vol. 36, no. 1, pp. 276–293, 2019. 10.1080/02656736.2018.1564155 30676101

[50] A. Makridis, K. Kazeli, P. Kyriazopoulos, N. Maniotis, T. Samaras, and M. Angelakeris, “An accurate standardization protocol for heating efficiency determination of 3D printed magnetic bone scaffolds,” J. Phys. D: Appl. Phys., vol. 55, no. 43, 2022, Art. no. 435002.

[51] M. B. Lodi , “Design and characterization of magnetic scaffolds for bone tumor hyperthermia,” IEEE Access, vol. 10, pp. 19768–19779, 2022.

[52] A. Makridis, S. Curto, G. V. Rhoon, T. Samaras, and M. Angelakeris, “A standardisation protocol for accurate evaluation of specific loss power in magnetic hyperthermia,” J. Phys. D: Appl. Phys., vol. 52, no. 25, 2019, Art. no. 255001.

[53] A. Farzin, M. Fathi, and R. Emadi, “Multifunctional magnetic nanostructured hardystonite scaffold for hyperthermia, drug delivery and tissue engineering applications,” Mater. Sci. Eng.: C, vol. 70, pp. 21–31, 2017. 10.1016/j.msec.2016.08.060 27770883

[54] F. D. Cojocaru, V. Balan, I. M. Popa, A. Munteanu, A. Anghelache, and L. Verestiuc, “Magnetic composite scaffolds for potential applications in radiochemotherapy of malignant bone tumors,” Medicina, vol. 55, no. 5, 2019, Art. no. 153. 10.3390/medicina55050153 PMC6572575 31108965

[55] S. Dong, Y. Chen, L. Yu, K. Lin, and X. Wang, “Magnetic hyperthermia–synergistic H_2_O_2_ self-sufficient catalytic suppression of osteosarcoma with enhanced bone-regeneration bioactivity by 3D-printing composite scaffolds,” Adv. Funct. Mater., vol. 30, no. 4, 2020, Art. no. 1907071.

[56] D. Tompkins, B. Partington, R. Steeves, S. Bartholow, and B. Paliwal, “Effect of implant variables on temperatures achieved during ferromagnetic hyperthermia,” Int. J. Hyperthermia, vol. 8, no. 2, pp. 241–251, 1992. 1573313 10.3109/02656739209021779

[57] S. A. Shah, M. Hashmi, S. Alam, and A. Shamim, “Magnetic and bioactivity evaluation of ferrimagnetic ZnFe_2_O_4_ containing glass ceramics for the hyperthermia treatment of cancer,” J. Magnetism Magn. Mater., vol. 322, no. 3, pp. 375–381, 2010.

[58] J. Wells , “Challenges and recommendations for magnetic hyperthermia characterization measurements,” Int. J. Hyperthermia, vol. 38, no. 1, pp. 447–460, 2021. 33730953 10.1080/02656736.2021.1892837

[59] A. Fanti, M. B. Lodi, and G. Mazzarella, “Enhancement of cell migration rate toward a superparamagnetic scaffold using LF magnetic fields,” IEEE Trans. Magn., vol. 52, no. 10, Oct. 2016, Art. no. 5200508.

[60] M. B. Lodi, A. Fanti, G. Muntoni, and G. Mazzarella, “A multiphysic model for the hyperthermia treatment of residual osteosarcoma cells in upper limbs using magnetic scaffolds,” IEEE J. Multiscale Multiphys. Comput. Tech., vol. 4, pp. 337–347, 2019.

[61] S. Oh and C. M. Collins, “Experimental temperature and specific absorption rate mapping using MRI in a transmit-receive head coil at 3.0 t,” in Proc. Prog. Electromagn. Res. Symp. Abstr., 2008, pp. 429–430.

[62] H. Kato , “Development of a phantom compatible for MRI and hyperthermia using carrageenan gel-relationship between dielectric properties and NaCl concentration,” Int. J. Hyperthermia, vol. 20, no. 5, pp. 529–538, 2004. 15277025 10.1080/0265673042000199277

[63] R. A. Jaime, R. L. Basto, B. Lamien, H. R. Orlande, S. Eibner, and O. Fudym, “Fabrication methods of phantoms simulating optical and thermal properties,” Procedia Eng., vol. 59, pp. 30–36, 2013.

[64] C. Dichtl, P. Sippel, and S. Krohns, “Dielectric properties of 3D printed polylactic acid,” Adv. Mater. Sci. Eng., vol. 2017, 2017, Art. no. 6913835.

[65] L. M. Bollig, M. V. Patton, G. S. Mowry, and B. B. Nelson-Cheeseman, “Effects of 3-D printed structural characteristics on magnetic properties,” IEEE Trans. Magn., vol. 53, no. 11, Nov. 2017, Art. no. 2300806.

[66] J. Laureto, J. Tomasi, J. A. King, and J. M. Pearce, “Thermal properties of 3-D printed polylactic acid-metal composites,” Prog. Additive Manuf., vol. 2, no. 1, pp. 57–71, 2017.

[67] J. Sorocki, I. Piekarz, and M. Bozzi, “Broadband permittivity and permeability extraction of 3-D-printed magneto-dielectric substrates,” IEEE Microw. Wireless Compon. Lett., vol. 31, no. 10, pp. 1174–1177, Oct. 2021.

[68] R. Hergt, S. Dutz, R. Müller, and M. Zeisberger, “Magnetic particle hyperthermia: Nanoparticle magnetism and materials development for cancer therapy,” J. Phys.: Condens. Matter, vol. 18, no. 38, 2006, Art. no. S2919.

